# Coinfection with SARS-CoV-2 and Influenza A Virus Increases Disease Severity and Impairs Neutralizing Antibody and CD4^+^ T Cell Responses

**DOI:** 10.1128/jvi.01873-21

**Published:** 2022-03-23

**Authors:** Eun-Ha Kim, Thi-Quyen Nguyen, Mark Anthony B. Casel, Rare Rollon, Se-Mi Kim, Young-Il Kim, Kwang-Min Yu, Seung-Gyu Jang, Jihyun Yang, Haryoung Poo, Jae U. Jung, Young Ki Choi

**Affiliations:** a College of Medicine and Medical Research Institute, Chungbuk National University, Cheongju, Republic of Korea; b Infectious Disease Research Center, Korea Research Institute of Bioscience and Biotechnology, University of Science and Technology, Daejeon, Republic of Korea; c Department of Cancer Biology and Global Center for Pathogens Research and Human Health, Lerner Research Institute, Cleveland Clinic, Cleveland, Ohio, USA; d Center for Study of Emerging and Re-emerging Viruses, Korea Virus Research Institute, Institute for Basic Science (IBS), Daejeon, Republic of Korea; e Vinmec Research Institute of Stem Cell and Gene Technology, Vinmec Healthcare System, Hanoi, Vietnam; Emory University School of Medicine

**Keywords:** SARS-CoV-2, influenza A virus, coinfection, hACE2 mice, neutralizing antibody, T cell, cytokine, chemokine, bronchoalveolar lavage fluid, immune response

## Abstract

Given the current coronavirus disease 2019 (COVID-19) pandemic, coinfection of severe acute respiratory syndrome coronavirus 2 (SARS-CoV-2) and influenza A virus (IAV) is a major concern for public health. However, the immunopathogenic events occurring with coinfections of SARS-CoV-2 and IAV remain unclear. Here, we report the pathogenic and immunological consequences of SARS-CoV-2 and IAV H1N1 coinfection in the K18-hACE2 transgenic mouse model. Compared with a single infection with SARS-CoV-2 or IAV, coinfections not only prolonged the primary virus infection period but also increased immune cell infiltration and inflammatory cytokine levels in bronchoalveolar lavage fluid leading to severe pneumonia and lung damage. Moreover, coinfections caused severe lymphopenia in peripheral blood, resulting in reduced total IgG, neutralizing antibody titers, and CD4^+^ T cell responses against each virus. This study sheds light on the immunopathogenesis of SARS-CoV-2 and IAV coinfection, which may guide the development of effective therapeutic strategies for the treatment of patients coinfected with these viruses.

**IMPORTANCE** The cocirculation of influenza virus merging with the COVID-19 pandemic raises a potentially severe threat to public health. Recently, increasing numbers of SARS-CoV-2 and influenza virus coinfection have been reported from many countries. It is a worrisome issue that SARS-CoV-2 coinfection with other pathogens may worsen the clinical outcome and severity of COVID-19 and increase fatality. Here, we evaluated SARS-CoV-2 and IAV coinfection using the K18-hACE2 mouse model. Coinfected mice exhibited increased mortality with prolonged IAV shedding. Furthermore, coinfected mice showed a higher level of cytokines and chemokines than a single infection condition. Interestingly, our data show that coinfected mice showed significantly fewer virus-specific and neutralizing antibodies than the mice with a single infection. Overall, this study suggests that coinfection aggravates viral pathology by impaired neutralizing antibody response.

## INTRODUCTION

The coronavirus disease 2019 (COVID-19) pandemic caused by severe acute respiratory syndrome coronavirus 2 (SARS-CoV-2) has resulted in more than 238 million confirmed cases and nearly 4.8 million deaths worldwide as of December 2021 (1). The fatality rate of this disease is approximately 2.1%, with variable clinical manifestations ranging from asymptomatic to severe (1, 2). Even though considerable effort has been made to develop vaccines and therapeutic drugs for COVID-19, the numbers of cases and deaths are continuously increasing. In addition, the recent emergence of new variants of the SARS-CoV-2, such as the B.1.1.7 (alpha) lineage from the United Kingdom, B.1.351 (beta) lineage from South Africa, and P.1 (gamma) lineage from Brazil, and the B.1.617.2 (delta) lineage from India have the potential to make the pandemic worse. Since those variants harbor various mutations in their spike proteins, they may cause difficulties with current strategies of diagnosis (3) as well as vaccine programs (4).

Seasonal influenza A virus (IAV) infections could stand to compound the challenges and threats posed to public health by the coronavirus pandemic, especially in the winter season. Coinfection of IAV with other respiratory viruses has been reported (5), and such concomitant infections are thought to be one of the main causes of death during the influenza pandemic of the 20th century (5). Thus, the probable emergence of SARS-CoV-2 and IAV coinfection may pose a serious public health threat. Despite several reports of SARS-CoV-2 and IAV coinfection in many countries (6–9), social distancing measures seemed to lessen the impact of coinfection (10). However, with the loosening of public gatherings and travel restrictions, a much higher rate of coinfection with IAV this coming winter would likely occur.

While recent clinical studies suggest that the coinfection of SARS-CoV-2 with influenza virus results in a more severe disease manifestation in humans (11, 12), the immunopathogenic mechanisms are still largely unknown. Therefore, in this study, we utilized K18-hACE2 transgenic mice to evaluate the virological and immunological effects of SARS-CoV-2 with IAV coinfection. Our results demonstrate that coinfection of SARS-CoV-2 and IAV enhances disease severity with prolonged viral persistence of both viruses in the lungs, resulting in severe pneumonia and lung injury. Most surprisingly, coinfection induced lymphopenia leading to impaired adaptive immune responses, resulting in a reduced level of neutralizing antibody (NAb) and T cell responses.

## RESULTS

### Coinfection of SARS-CoV-2 and IAV enhances disease severity.

**FIG 1:**
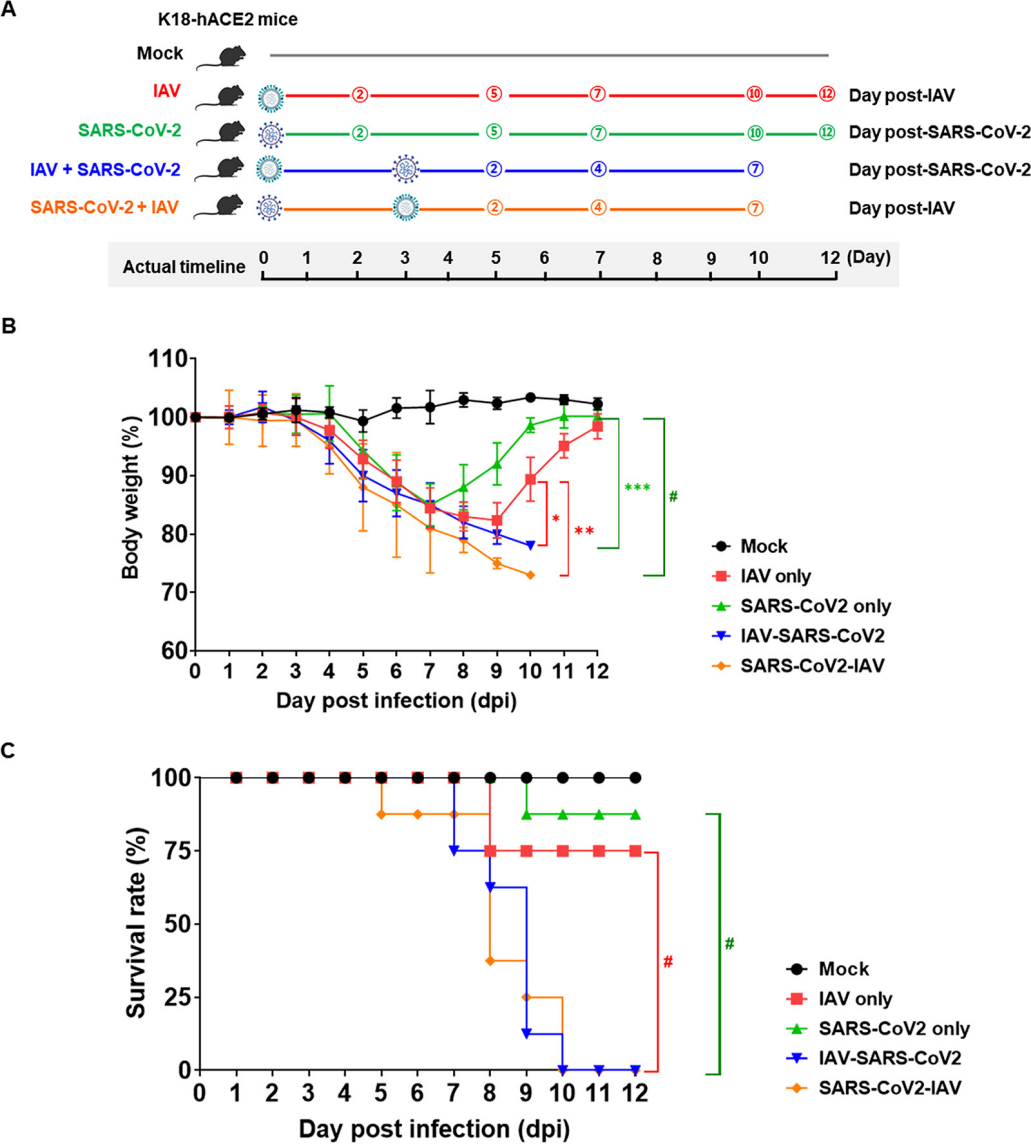
Coinfection of SARS-CoV-2 and IAV enhances disease severity. (A) Schematic diagram of experimental design. K18-hACE2 mice were infected with either IAV or SARS-CoV-2 on day 0. After 3 days, the mice were infected with the other virus for coinfection groups. Mice inoculated with PBS were used as the mock control. (B and C) Body weight (B) and survival (C) of mice were monitored for 12 days postinfection (dpi). The data are presented as mean ± SEM. Statistical significance was determined by two-way ANOVA/Tukey test for body weight change and log-rank test for survival rate; *, *P < *0.05; **, *P < *0.01; ***, *P < *0.001; *#*, *P < *0.0001. Red asterisks indicate statistical significance between IAV single infection, and IAV+SARS-CoV-2 and SARS-CoV-2+IAV coinfection, and green asterisk indicates statistical significance between SARS-CoV-2 single infection, and IAV+SARS-CoV-2 and SARS-CoV-2+IAV coinfection at 10 dpi.

To investigate whether coinfection of SARS-CoV-2 with IAV could affect disease severity, groups of K18-hACE2 mice (*n* = 8) were infected with 30 μL of 10^4.0^ 50% tissue culture infective dose (TCID_50_/mL) of the pandemic IAV H1N1 (A/California/04/09) or 10^5.5^ TCID_50_/mL of SARS-CoV-2 (NMC-nCoV02) through the intranasal route (Fig. 1A). Infection groups include mock-phosphate-buffered saline (PBS), IAV only, SARS-CoV-2 only, IAV infection prior to SARS-CoV-2 infection (IAV + SARS-CoV-2), and SARS-CoV-2 infection prior to IAV infection (SARS-CoV-2 + IAV), with a 3-day interval between infections in the coinfection groups. Infected mice were monitored for body weight and survival rate for 12 days. While PBS-infected mice maintained their body weight with 100% survivability, the groups of virus-infected mice showed a continuous body weight reduction from 4 days postinfection (dpi) (Fig. 1B). While the SARS-CoV-2 single-infection group exhibited a rapid decline of body weight until 7 dpi with an 87.5% survival rate, the IAV single-infection mice showed continuous and severe body weight loss until 9 dpi with a 75% survival rate (Fig. 1B and C). Interestingly, coinfection groups exhibited significantly lower survival rates and body weight than the single-infection groups (Fig. 1B and C). Notably, both coinfection groups caused 100% mortality in mice at 10 dpi (Fig. 1C). These results suggest that the coinfections of SARS-CoV-2 and IAV could significantly increase disease severity and fatality compared with a single infection with either virus.

### Coinfection of SARS-CoV-2 and IAV prolongs virus infection in lungs and BALF.

**FIG 2:**
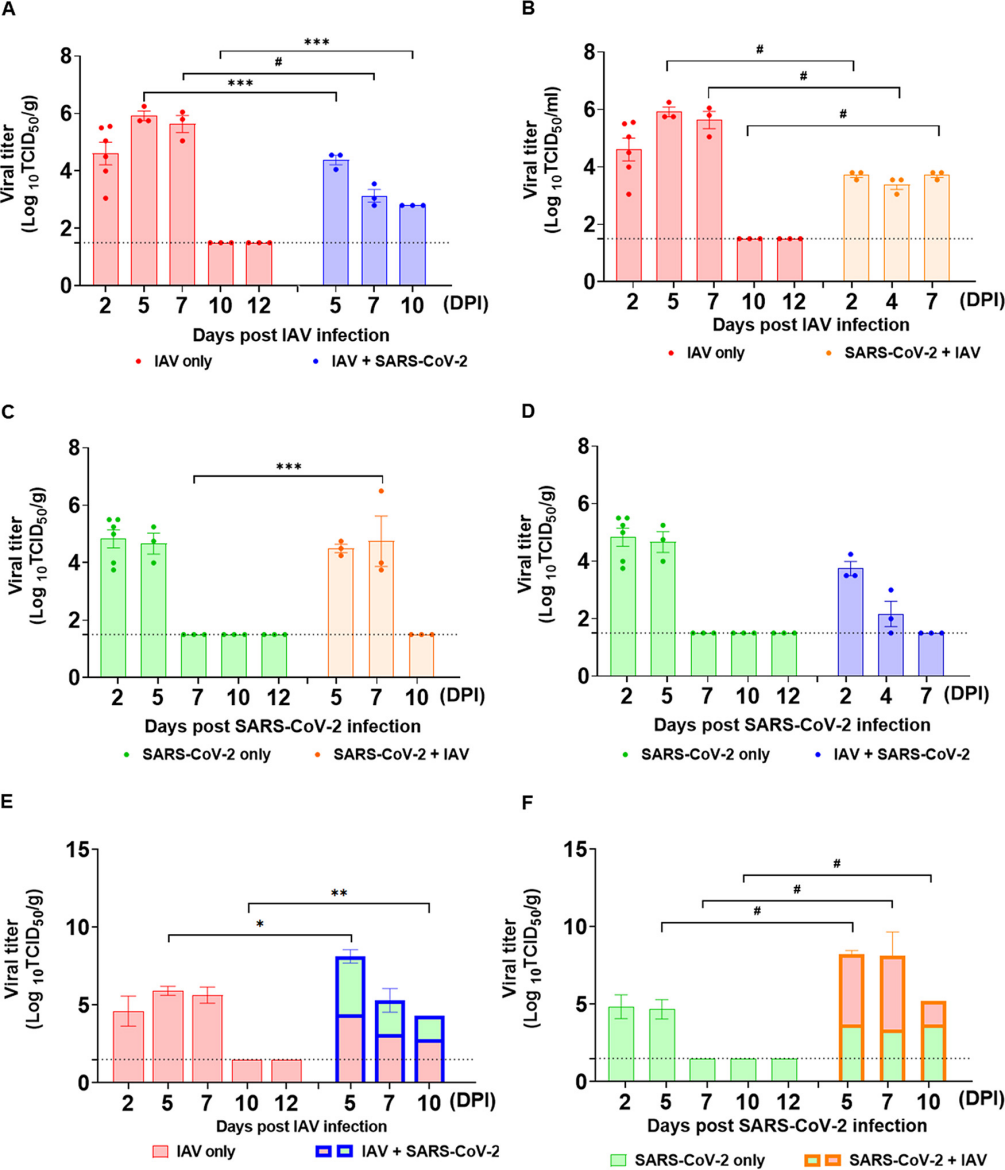
Coinfection of SARS-CoV-2 and IAV prolongs virus persistence in lungs. K18-hACE2 mice were infected with either IAV or SARS-CoV-2 on day 0, and for coinfection groups, mice were infected with the other virus at 3 days post-primary infection. Infectious IAV virus titers (A and B) and infectious SARS-CoV-2 titers (C and D) were measured in lungs of infected mice at 2, 5, 7, 10, and 12 dpi in single-infection groups and post-primary virus infection in coinfection groups (while 2, 4, and 7 dpi postsecondary infection in coinfection groups would represent the 5, 7, and 10 dpi). (E and F) Total viral load (sum of infectious IAV and SARS-CoV-2) in lungs at the indicated time points. Data are shown as log_10_TCID_50_ and presented as mean ± SEM. Dashed lines indicate the limit of detection. Statistical significance was determined by two-way ANOVA/Tukey; *, *P < *0.05; **, *P < *0.01; ***, *P < *0.001; *#*, *P < *0.0001.

To assess whether coinfection of SARS-CoV-2 and IAV affects viral titers of the primary or secondary infected virus in mice, lungs and BALF were collected from each infection group (*n* = 3) at 2, 5, 7, 10, and 12 dpi in single-infection groups and after primary virus infection in coinfection groups (while 2, 4, and 7 dpi postsecondary infection in coinfection groups would represent the 5, 7, and 10 dpi) for virus titer determination using Madin-Darby canine kidney (MDCK) cells or Vero cells. Mice infected with IAV maintained only high infectious virus titers in the lung and bronchoalveolar lavage fluid (BALF) until 5 dpi (Fig. 2 and Fig. S1 in the supplemental material), whereas infectious IAV titers persisted until 10 dpi in IAV + SARS-CoV-2 coinfection mice lung tissues (Fig. 2A). However, it is noteworthy that IAV titers in SARS-CoV-2 + IAV coinfection group were lower than those of a single-IAV infection group at all time points (Fig. 2B).

The SARS-CoV-2 single-infection group showed the highest virus titer at 2 dpi (4.83 log_10_ TCID_50_/g), which was maintained until 5 dpi, and the infectious virus was not detected from 7 until 12 dpi in the lungs (Fig. 2C). However, SARS-CoV-2 + IAV coinfection group showed a prolonged persistence of SARS-CoV-2 until 7 dpi with titers as high as 4.75 log_10_ TCID_50_/g (Fig. 2C). Conversely, there is an observable lower SARS-CoV-2 titer in the IAV + SARS-CoV-2 coinfection group than that in the SARS-CoV-2 single-infection group (Fig. 2D and Fig. S1). These results reveal that the primary infected virus persists longer in the lungs and BALF during coinfection, while replication of the secondarily infected virus is shown to have reduced growth kinetics compared with the primary virus infection. These findings raise a question of how both coinfection groups exhibit 100% mortalities in 10 days even with the attenuated virus titers of the secondary virus in the coinfection groups. To address this question, we assessed the combined titers of IAV and SARS-CoV-2 to present total virus loads in the mouse lungs. The results revealed that although the secondary SARS-CoV-2 or IAV infection showed attenuated virus titers during coinfection, the compounding viral loads (SARS-CoV-2 + IAV) in coinfected mice are higher than that in a single infection (Fig. 2E and F).

### Coinfection of SARS-CoV-2 and IAV causes unbalanced immune responses in the lung and peripheral blood.

**FIG 3:**
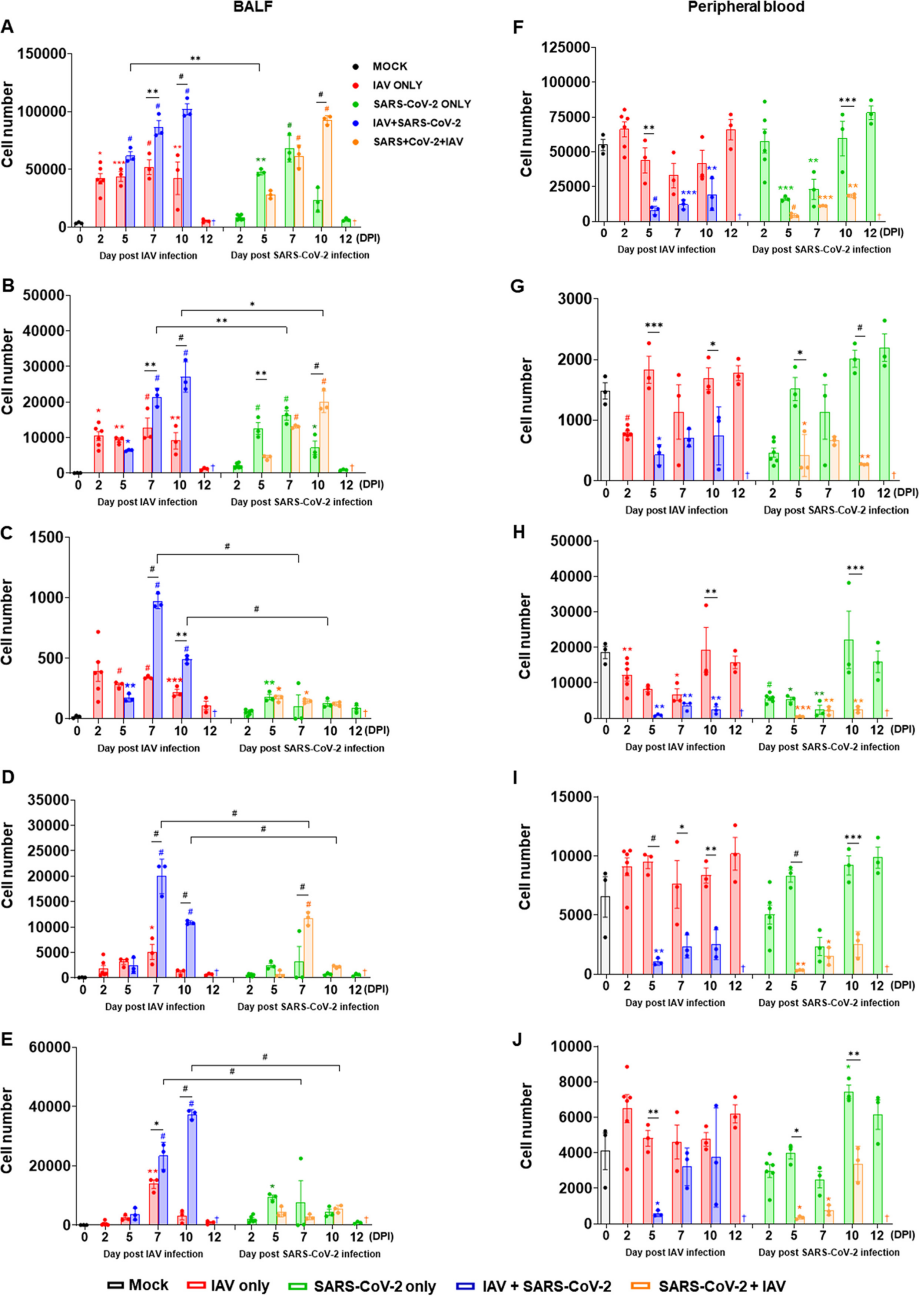
Coinfection of SARS-CoV-2 and IAV results in excessive immune cell recruitment into the BALF, while also causing lymphopenia in peripheral blood. K18-hACE2 mice were infected with either IAV or SARS-CoV-2 on day 0. For coinfection groups, mice were infected with the second virus 3 days after the primary infection. Results of analysis of immune cell populations in mice BALF (A to E), including CD45^+^ cells (A), monocytes (B), B cells (C), CD4^+^ T cells (D), and CD8^+^ T cells (E) and in peripheral blood (F to J), including CD45^+^ cells (F), monocytes (G), B cells (H) CD4^+^ T cells (I),and CD8^+^ T cells (J) at 2, 5, 7, 10, and 12 dpi post-primary virus infection. Data are presented as mean ± SEM. Statistical significance was determined by two-way ANOVA/Tukey; ^#^, indicates the comparison between indicated groups; *, comparison with mock group (0 dpi); *, *P < *0.05; **, *P < *0.01; ***, *P < *0.001; *#*, *P < *0.0001. †, the experiment was not performed because no mice survived. Each of the four colored asterisk/octothorpe (red, blue, green, and orange) indicates statistical significance compared to mock group (0 dpi).

Excessive inflammatory responses are associated with severe pulmonary pathophysiological conditions upon influenza or SARS-CoV-2 infection (2, 13). To evaluate the inflammatory responses during viral infections, we performed flow cytometric analysis using BALF from each set of virus-infected mouse groups. Immune cell infiltration analysis revealed that most of the virus-infected mice have significantly higher numbers of CD45^+^ cells than the PBS-infected mice (Fig. 3A). The IAV single-infection group showed a rapid increase of CD45^+^ cells in BALF as early as 2 dpi that was maintained until 10 dpi (Fig. 3A, left). Although a relatively lower CD45^+^ cell infiltration in the SARS-CoV-2 single-infection group than that in the IAV single-infection group was observed at 2 dpi, the SARS-CoV-2 single-infection group showed a rapid increase in the CD45^+^ cell numbers in the BALF from 5 dpi (Fig. 3A, right). Furthermore, the IAV + SARS-CoV-2 coinfection group showed a rapid increase of CD45^+^ cells in BALF from 7 dpi compared with the IAV single-infection group and continuously increased until 10 dpi. However, the SARS-CoV-2 + IAV coinfection group showed relatively slower CD45^+^ cell infiltration than the IAV + SARS-CoV-2 coinfection group, although both coinfection groups ultimately reached a similar level at 10 dpi. Similarly, the SARS-CoV-2 single-infection group showed slower kinetics of monocytes and B cells infiltrations than the IAV single-infection group at 2 dpi, but the SARS-CoV-2 single-infection group showed even higher cell numbers at 5 dpi (Fig. 3B and C, right). On the other hand, there was no significant difference in the kinetics of CD4^+^T and CD8^+^T cell infiltrations in BALF between the IAV and SARS-CoV-2 single-infection groups (Fig. 3D and E). However, it is noteworthy that the IAV + SARS-CoV-2 coinfection group showed much higher monocytes, B cells, CD4^+^T cells, and CD8^+^ T cells infiltrations in BALF than the SARS-CoV-2 + IAV coinfection group at 7 and 10 dpi. These results suggest that the SARS-CoV-2 infection may cause a delayed immune cell infiltration into the lungs compared with IAV infection.

Recent studies have reported that lymphopenia is one of the characteristic markers of severe COVID-19 (14). To investigate the impact of each virus infection on immune cell populations, peripheral blood samples were collected from each infection group and the populations of CD45^+^ cells, monocytes, CD4^+^ T cells, CD8^+^ T cells, and B cells were assessed by flow cytometry (Fig. 3F to J). The IAV single infection does not cause significant cell number variation in the peripheral blood, while the SARS-CoV-2 single-infection group showed significantly lower CD45^+^ cells (at 5 and 7 dpi) (Fig. 3F, right), monocytes (at 2 dpi) (Fig. 3G, right), and B cells (at 7 dpi) (Fig. 3H, right) than the PBS-treated control group. However, no significant alteration was observed in CD4^+^ and CD8^+^ T cell numbers by either of the single-virus infections (Fig. 3I and J). Surprisingly, in the coinfection groups, most of the immune cells tested were significantly lower than the PBS control group, especially in the CD45^+^ cells, monocytes, B cells, and CD4^+^ T cell numbers, which were significantly lower at all time points than those of the PBS control group (Fig. 3F to J). Furthermore, most tested immune cell populations in the peripheral blood of coinfections were significantly lower than those in both the single-infection groups except CD8^+^ T cells which rapidly recovered the cell numbers from 7 and 10 dpi from IAV + SARS-CoV-2 and SARS-CoV-2 + IAV infection, respectively. Taken together, these results demonstrate that IAV and SARS-CoV-2 coinfection leads to severe lymphopenia in peripheral blood.

### Increased severity of lung damage following SARS-CoV-2 and IAV coinfection.

**FIG 4:**
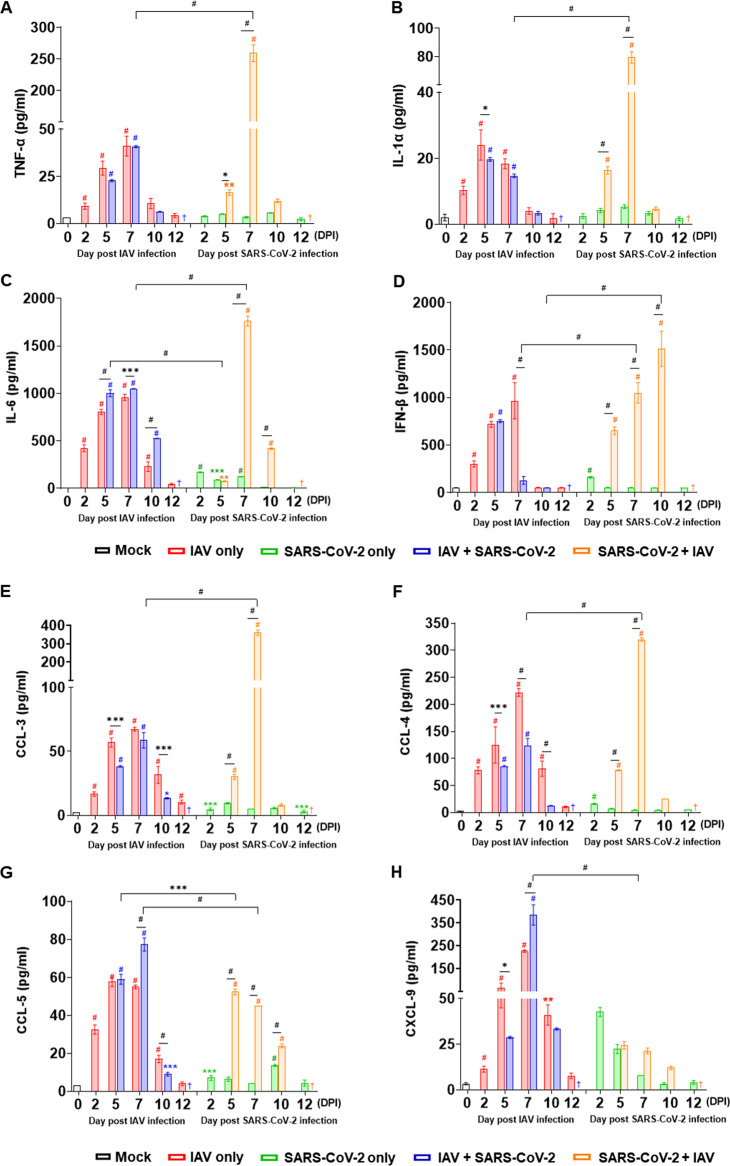
Coinfection of SARS-CoV-2 and IAV increases lung injury. K18-hACE2 mice were infected with either IAV or SARS-CoV-2 on day 0. For coinfection groups, 3 days after primary infection, mice were infected with the second virus. Levels of inflammatory cytokines (A to D), including TNF-α (A), IL-1α (B), IL-6 (C), and IFN-β (D), in mice BALF at 2, 5, 7, 10, and 12 dpi post-primary virus infection are shown. Levels of inflammatory chemokines (E to H), including CCL3 (E), CCL4 (F), CCL5 (G) and CXCL9 (H), in mice BALF at 2, 5, 7, 10 and 12 dpi post-primary virus infection are shown. Data are presented as mean ± SEM. Statistical significance was determined by two-way ANOVA/Tukey; *, *P < *0.05; **, *P < *0.01; ***, *P < *0.001; *#*, *P < *0.0001. †, the experiment was not performed because no mice survived. Each of the four colored asterisk/octothorpe (red, blue, green, and orange) indicates statistical significance compared to mock group (0 dpi).

To determine whether the coinfection of IAV and SARS-CoV-2 affects cytokine production, a set of proinflammatory cytokines, including tumor necrosis factor alpha (TNF-α), interleukin-1α (IL-1α), IL-6, and interferon beta (IFN-β), were measured using BALF from the infected mice. The IAV single-infection group showed a rapid increase in the levels of TNF-α, IL-1α, IL-6, and IFN-β from 2 dpi to 7 dpi (Fig. 4A to D). While the SARS-CoV-2 single-infection group showed moderate increases of IL-1α (at 7 dpi), IL-6 (at 2, 5, 7 dpi), and IFN-β (2 dpi), their expression levels were lower than those of the IAV single-infection group (Fig. 4A to D). Interestingly, the IAV infection followed by secondary SARS-CoV-2 infection showed negligible increases of inflammatory cytokines in BALF compared with the IAV single-infection group (except IL-6; 10 dpi). However, SARS-CoV-2 infection followed by IAV coinfection exhibited rapid increases of TNF-α, IL-1α, IL-6, and IFN-β levels (Fig. 4A to D). Furthermore, the total amount of the cytokine expressions was significantly higher in the SARS-CoV-2 + IAV group than that in the IAV + SARS-CoV-2 coinfection group at 7 dpi, suggesting differential mechanisms are involved in the regulation of the cytokine production depending on the timing of viral coinfection. Notably, while the level of IFN-β was decreased markedly by secondary SARS-CoV-2 infection at 7 dpi in the IAV + SARS-CoV-2 coinfection group, the opposite patterns were observed in the SARS-CoV-2 + IAV group from 5 to 10 dpi (Fig. 4D).

Similarly, while SARS-CoV-2 infection alone induced a minimal expression of the inflammatory chemokines, the IAV single-infection group induced significantly high levels of CCL3, CCL4, CCL5, and CXCL9. It is noteworthy that while the CCL3 and CCL4 levels were significantly increased in SARS-CoV-2 + IAV at 7 dpi (Fig. 4E and F, right), the CCL5 and CXCL9 were significantly higher in IAV + SARS-CoV-2 coinfection groups at 7 dpi than those of the SARS-CoV-2 + IAV group (Fig. 4G and H), suggesting potential differential immune responses by order of viral infections.

Moreover, lung histopathological data further illustrate that coinfections induce more immune cell infiltration with severe pathological regions than either of the single infections (see Fig. S2 in the supplemental material). Taken together, these results show that SARS-CoV-2 and IAV coinfection induces higher immune cell recruitment to the lungs than either single infection by mediating chemokine production leading to a serious lung injury.

### Severe lymphopenia in coinfected mice causes reduced neutralizing antibody and T cell responses.

**FIG 5:**
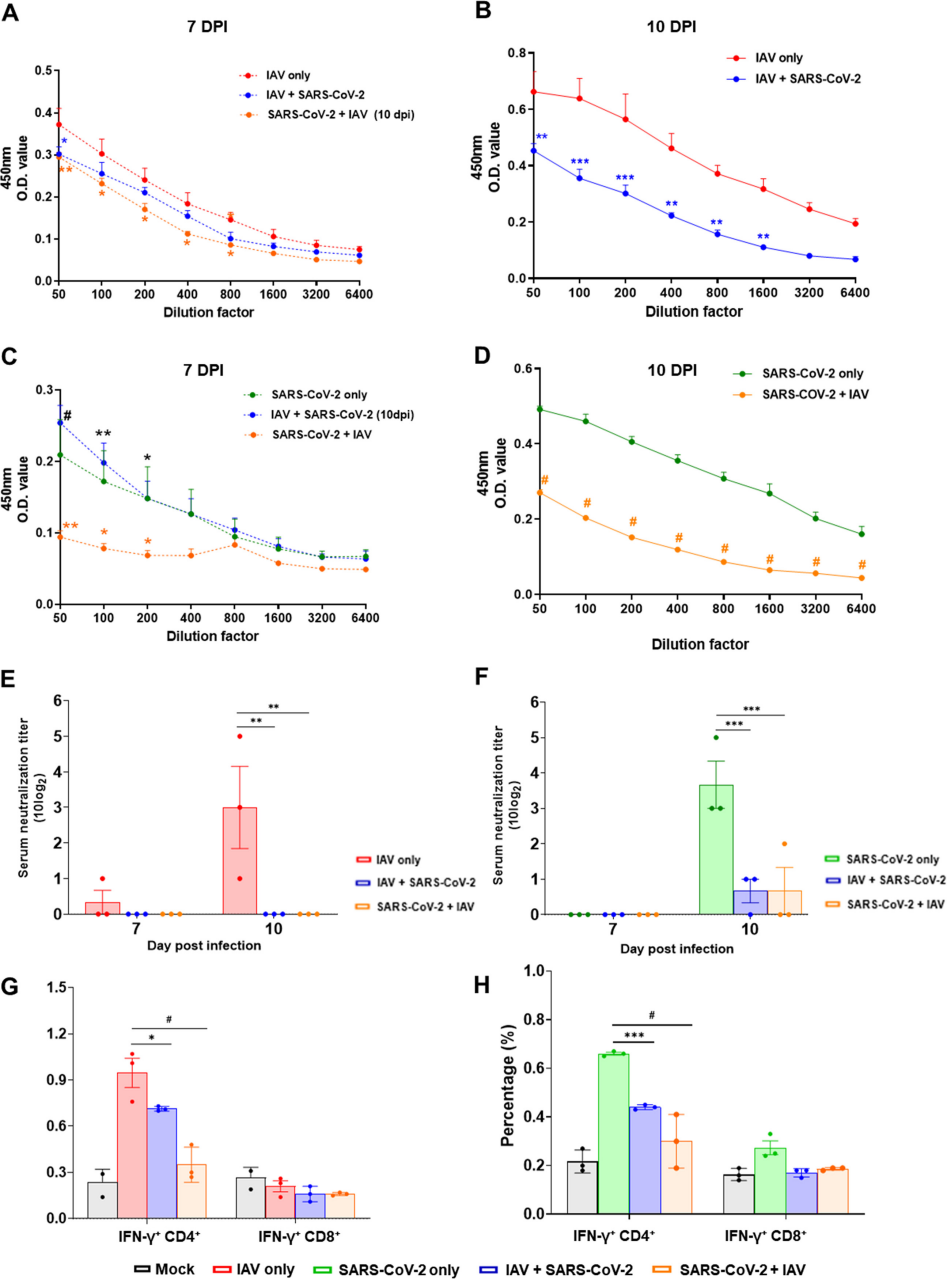
Coinfection of SARS-CoV-2 and IAV reduces total IgG levels, neutralizing antibody titers, and CD4^+^ T cell response. K18-hACE2 mice were infected with either IAV or SARS-CoV-2 on day 0. For coinfection groups, 3 days after primary infection, mice were infected with the second virus. Total IgG levels against IAV (A and B) and SARS-CoV-2 (C and D) and neutralizing Abs against IAV (E) and SARS-CoV-2 (F) were measured in sera collected from each group of mice at 7 and 10 dpi post-primary virus infection (while 7 dpi postsecondary infection in coinfection groups would represent the 10 dpi). Data are presented as geometric mean ± SEM. Frequencies of IFN-γ-positive CD4^+^ and CD8^+^ T cells specific to IAV (G) and SARS-CoV-2 (H) were measured in splenocytes of the infected mice at 10 dpi post-primary virus infection. Statistical significance was determined by two-way ANOVA/Tukey; *, *P < *0.05; **, *P < *0.01; ***, *P < *0.001; *#*, *P < *0.0001. (A to D) Blue asterisk/octothorpe and orange asterisk/octothorpe indicate statistical significance in comparison to the single-infection group.

Given that the numbers of peripheral B and CD4 T cells were dramatically reduced by coinfections (Fig. 3H and I), we next monitored the level of total IgG and serum neutralizing antibody (NAb) in each group of mice at 7 and 10 dpi from the primary virus infection. The IAV single infection group showed a significantly higher level of IAV virus-specific IgG titers than that of either coinfection group at 7 and 10 dpi in survived mice (Fig. 5A and B). Similarly, higher IgG titers against SARS-CoV-2 were observed in single SARS-CoV-2 infected mice than that of SARS-CoV-2 + IAV coinfection mice at 7 and 10 dpi (Fig. 5C and D). Interestingly, IAV single infection group induced strong NAb titers against IAV at 10 dpi, but both coinfection groups showed nearly undetectable NAbs against IAV at both 7 and 10 dpi (Fig. 5E). Furthermore, the SARS-CoV-2 single infection group exhibited the high NAb titers at 10 dpi against SARS-CoV-2 virus, while both coinfection groups showed considerably lower NAb against the SARS-CoV-2 at 10 dpi (Fig. 5F). These results suggest that severe lymphopenia induced by SARS-CoV-2 and IAV coinfection is strongly associated with the impaired production of IgG and NAbs against SARS-CoV-2 and IAV.

Finally, to investigate whether the decrease in peripheral T cell population in coinfected mice could affect antiviral T cell responses against IAV and SARS-CoV-2, the level of IFN-γ in CD4^+^ and CD8^+^ T cells were measured by intracellular staining using the spleen from the survived mice at 10 dpi. Our results revealed that the percentage of the IAV-specific IFN-γ positive CD4^+^ T cells was higher in the IAV single infection group (0.95 ± 0.1%) compared to that of IAV + SARS-CoV-2 (0.71 ± 0.01%, *P* = 0.018) and SARS-CoV-2 + IAV (0.35 ± 0.06, *P* < 0.0001) coinfection groups (Fig. 5G). However, there were no statistical differences in IAV-specific IFN-γ positive CD8^+^ T cells among the groups tested (Fig. 5G). Similarly, a significantly higher percentage of SARS-CoV-2-specific IFN-γ positive CD4^+^ T cells was also observed in the SARS-CoV-2 single infection group (0.66 ± 0.01%) than in the IAV + SARS-CoV-2 (0.44 ± 0.01%, *P* < 0.001) and SARS-CoV-2 + IAV (0.30 ± 0.06, *P* < 0.0001) coinfection groups. However, there was no statistical difference in SARS-CoV-2-specific IFN-γ positive CD8^+^ T cells among the groups (Fig. 5H). Altogether, these data suggest that the coinfection of SARS-CoV-2 and IAV significantly impairs the CD4^+^ T cell responses against each virus.

## DISCUSSION

Coinfection of viruses has been frequently occurring in nature (15). Recently, with the rapid spread of SARS-CoV-2, cases of coinfections with influenza viruses have been continuously reported with the speculation that concomitant infection with these two viruses causes more severe illness (6, 9, 14, 16–20). Although experimental data regarding SARS-CoV-2 and IAV coinfection has been shown to cause more severe disease in the golden Syrian hamster and transgenic mouse models (21–23), however, the investigation was only limited to analyses of disease outcomes, such as mortality. Even though consequences of coinfections may vary depending on various factors, such as timing between infections, virus dose, route of infection, and the age (15). Herein, we adapted the K18-hACE2 mice model and investigated the underlying virological and immunological impact of coinfection of influenza A virus and SARS-CoV-2 (IAV infection before SARS-CoV-2 infection and vice versa) in comparison to a single infection with these viruses. Our results demonstrate that coinfection of SARS-CoV-2 and IAV lead to an enhanced body-weight loss with an increased mortality rate in K18-hACE2 mice compared to the single infection groups. Results also show a prolonged presence of the virus in the lungs, together with the increased levels of inflammatory cytokines and chemokines leading to increased lung injury. Moreover, SARS-CoV-2 and IAV coinfection is accompanied by severe lymphopenia which leads to reduced antibody and CD4^+^T cell responses against each virus.

It is noteworthy that secondary infection with SARS-CoV-2 prolonged the primary IAV infection up to 10 dpi in the lungs and BALF, and likewise, secondary infection with IAV prolonged SARS-CoV-2 persistence up to 7 dpi in both the lung and BALF. However, both coinfection groups exhibited that secondarily infected virus has suppressed replication in the lungs and BALF compared with the single virus infection groups. These data are consistent with previous studies in which primary infection with rhinovirus inhibits influenza infection (24), and coinfection with IAV 1 day prior to SARS-CoV-2 was found to result in lower lung SARS-CoV-2 titers than a single infection with SARS-CoV-2 in golden Syrian hamster models (23). These observations are thought to be due to viral interference, the most common outcome of coinfections involving viruses (15). However, our results demonstrate that the compounded viral loads (both IAV plus SARS-CoV-2 titers) in lungs and BALF in coinfection groups were much higher than with single virus infections. Furthermore, lung pathology was observed in all infected groups, while coinfected mice exhibited a more severe pathological outcome compared to any of the single infection. Albeit difficult to compare the pathological manifestation between coinfection to those in single infection groups at 7 dpi, a possible explanation for the increased lung pathological manifestation might be the exacerbated immune and pathological responses activated by the secondary virus infection, which may explain the increased mortality in both coinfected mouse groups.

The development of immunomodulatory strategies to treat cases of SARS-CoV-2 and IAV coinfection requires a fundamental understanding of the involved immune responses. Rapid and transient immune cell recruitment to inflamed tissues is a natural response to foreign pathogens. However, excessive or prolonged cell extravasation can induce tissue damage due to the production of disproportionate inflammatory cytokines, chemokines, and reactive oxygen species by the cells (25). Moreover, infection with IAV or SARS-CoV-2 alone has been demonstrated to cause excessive inflammatory responses (2, 13). In this study, we found that coinfection of SARS-CoV-2 and IAV enhanced the recruitment of CD45^+^ cells, monocytes, and T cells into the lungs compared to the single infection groups, suggesting that coinfection could exacerbate upregulated cell recruitment induced by a single infection. It is noteworthy that the overall cytokine expression levels were significantly lower in the SARS-CoV-2 infection group than those in the IAV single infection group which may closely be associated with overall inhibition of IFN-β by SARS-CoV-2 infection. Further, the secondary SARS-CoV-2 infection in IAV + SARS-CoV-2 group resulted in a cytokine profile similar to that of infection with IAV alone, while the secondary infection with IAV in SARS-CoV-2 + IAV group induces a marked increase of inflammatory cytokines, including TNF-α, IL-1α, IL-6, and IFN-β in BALF suggesting differential immune responses by order of viral infections. It is reported that the excessive production of inflammatory cytokines, such as TNF-α and IL-1α, induces disseminated intravascular coagulation, respiratory failure, and multiorgan failure (26). Furthermore, IL-6 was significantly increased in mice sera coinfected with SARS-CoV-2 and IAV. IL-6 signaling was shown to play a crucial role in vascular endothelial cell dysfunction in cytokine release syndrome and the progression of acute inflammatory diseases such as acute respiratory distress syndrome (27). Hence, increased serum IL-6 may be involved in the enhanced disease severity observed during coinfection. These data suggest that a targeting strategy such as immunosuppressive drugs to prevent excessive inflammatory responses might be beneficial for coinfection patients.

B cells secret antibodies to block virus infection, CD4^+^ T cell helps B cells to proliferate and produce antibodies, while cytotoxic T cells function to kill virus-infected cells. Thus, the induction of proper immune responses plays an essential role in combating viral infections. However, lymphopenia has been commonly reported in severe cases of COVID-19 (27). The decrease in lymphocytes would lead to more severe disease due to impaired adaptive immune responses. Further, recent studies reported that lymphopenia is observed in about 85% of critically ill COVID-19 patients (18, 28, 29) and about 71.4% of influenza-infected patients with poor outcomes (defined as respiratory failure, sepsis-related organ failure, or death) (30). Our results demonstrate significant decreases of B cells and CD4^+^ T cells in peripheral blood of coinfection compared to single-infected mice. While cell numbers in single infection groups returned to levels similar to those of PBS-treated mice at 10 and 12 dpi, cell numbers did not recover in coinfected mice. Nonetheless, the underlying mechanisms of lymphopenia caused by SARS-CoV-2 remain unclear. Possible mechanisms of lymphopenia by SARS-CoV-2 infection have been previously suggested such as (1) cytokine storm, (2) T cell exhaustion and cell death, and (3) inhibition of T cell expansion (27, 31). In addition, cell migration into inflamed tissues was also reported to be associated with lymphopenia in patients with severe COVID-19 (18, 32). A previous study reported that chemokines CCL3 and CXCL9 are involved in recruiting T and B cells to the pulmonary microenvironment in patients with severe COVID-19 (33). Further, CCL4, also known as Macrophage inflammatory protein-1β (MIP-1β) is a chemoattractant for natural killer cells, monocytes, and a variety of other immune cells (34). In this study, we found a remarkable elevation in CCL3, CCL4, and CXCL9 chemokine levels and an increased T and B cell count in BALF of coinfected groups. This finding suggests that peripheral lymphocytes might be chemotactically migrated into the inflamed tissues thereby contributing to the lymphopenia seen in peripheral blood. Furthermore, recent studies showed that SARS-CoV-2 RNA and IAV RNA were detected in both epithelial and immune cells, including T cells and plasma B cells resulting in cell death, which may coincide with lymphopenia (35, 36). Collectively, these findings could explain the mechanism regarding T and B cell reduction observed in IAV and SARS-CoV-2 coinfected mice.

There have been few studies on the critical role of B cells in COVID-19 pathogenesis, although neutralizing antibodies are known to play a critical role in the protection against SARS-CoV-2 infection (37) and transmission (38). Here, we found that coinfection of SARS-CoV-2 and IAV significantly reduced levels of total IgG and neutralizing antibodies against both IAV and SARS-CoV-2. A previous study reported that frequencies of SARS-CoV-2-specific IFN-γ secreting CD4^+^ T cells are lower in COVID-19 patients requiring intensive care, compared to those in recovered patients (39). Interestingly, we observed that frequencies of IAV- and SARS-CoV-2-specific IFN-γ positive CD4^+^ T cells were remarkably lower in coinfection groups than in single infection group, although the IAV- and SAR-CoV-2-specific IFN-γ positive CD8^+^ T cells were similar among both. With the results of lymphopenia in confection groups, the reduced NAb titers against both viruses correlate with decreased B and CD4^+^ T cell populations in peripheral blood, and thus the CD4^+^ T cell response could be more vital than CD8^+^ T cell response in protection against coinfection by NAb. However, in this study, we can only evaluate immune response until 10 dpi since the coinfected hACE2 mice model succumb within this time point. In addition, further studies regarding IFN-γ positive CD4^+^ and CD8^+^ T cells in the lungs and BALF is also necessary to elucidate T cell responses in the lungs.

Taken together, our data explicate the virological and immunological interactions between SARS-CoV-2 and IAV during the coinfection, suggesting coinfection with these viruses could lead to increased disease severity. This study could be of value for the rational development of therapeutic strategies for the effective treatment of coinfected patients and may shed some light apropos to immune pathogenesis during SARS-CoV-2 and IAV coinfection.

## MATERIALS AND METHODS

### Animal model and ethics statement.

K18-hACE2 mice were obtained from the Jackson Laboratory. The animals were housed and bred at Korea Research Institute of Bioscience and Biotechnology (Daejeon, South Korea) and maintained in isolators in a biosafety level 3 (BSL3) laboratory at Chungbuk National University (KCDC-14-3-07) during the experiments. Animal studies were performed according to experimental procedures approved by the Institutional Animal Care and Use Committee (IACUC) of Chungbuk National University (approval number CBNUR-1338-21).

### Cells lines.

Vero cells and Madin-Darby canine kidney (MDCK) cells were maintained in Dulbecco’s modified Eagle medium (DMEM) (Gibco) supplemented with 10% heat-inactivated fetal bovine serum (FBS) and 1% penicillin-streptomycin (PS) (Gibco) and Eagle’s minimum essential medium (EMEM) (HyClone) supplemented with heat-inactivated 10% FBS, 1% vitamins, and 1% PS for virus propagation.

### Virus infection of K18-hACE2 mice and sample collection.

NMC-nCoV02, a SARS-CoV-2 strain isolated from a COVID-19-confirmed patient in South Korea, was propagated in Vero cells and used for the animal infection study. Pandemic H1N1 influenza A virus (A/California/04/09) was provided generously by R.G. Webster (Division of Virology, Department of Infectious Diseases, St. Jude Children’s Research Hospital, Memphis, TN) and propagated in MDCK cells. For virus inoculation, 6- to 8-week-old (female) K18-hACE2 mice were divided into 5 groups (mock group, 11 mice; single infection group, 29 mice/group; coinfection group, 26 mice/group) as follows: mock-PBS, IAV only, SARS-CoV-2 only, IAV infection prior to SARS-CoV-2 infection (IAV + SARS-CoV-2), and SARS-CoV-2 infection prior to IAV infection (SARS-CoV-2 + IAV). The mice were anesthetized with isoflurane and then infected intranasally with the SARS-CoV-2 (10^5.5^ TCID_50_/mL, 30 μL) and/or IAV (10^4^ TCID_50_/mL, 30 μL). Body weight loss and survival rates were monitored for 12 days postinfection. Mice with more than a 25% decrease in body weight were euthanized humanely. Lungs, bronchoalveolar lavage fluid (BALF), and sera were collected from each group of mice after euthanasia (*n* = 3) at 2, 5, 7, 10, and 12 dpi in single-infection groups and after primary virus infection in coinfection groups (2, 4, and 7 dpi postsecondary infection in coinfection groups would represent the 5, 7, and 10 dpi) for further analysis. Viral infections and downstream experiments were performed in the BSL3 laboratory.

### Flow cytometric analysis.

To obtain cells from BALF, mice were sacrificed followed by cannulation of the trachea with a 20G catheter. BAL was performed with three washes of 0.8 mL sterile PBS. BALF was centrifuged, and cells were resuspended in PBS with 2% FBS. To obtain cells from peripheral blood, blood from infected mice was collected from the submandibular vein. Erythrocytes were lysed twice with red blood cell lysing buffer (Thermo Fisher Scientific, Waltham, USA), and the remaining cells were suspended in PBS with 2% FBS. All cells were preincubated with CD16/CD32 Fc blocking antibody (Ab) (BD Biosciences, San Diego, USA) for 10 min before staining. To assess myeloid cells, cells were stained with antibodies against the following markers: AF700 anti-CD45 (clone 30 F-11), APC-Cy7 anti-CD11c (clone N418), PE-Cy7 anti-Ly6G (clone 1A8), and BV605 anti-Ly6C (clone HK1.4). To examine the proportions of lymphoid cells, cells were stained with antibodies against the following markers: AF700 anti-CD45 (clone 30 F-11), APC anti-CD3 (clone 17A2), anti-CD4 (clone RM4-5), and PE-Cy7 anti-CD8 (clone 53-6.7) (Biolegend). After staining, all cells were washed and stained with fixable viability Dye eFluor 780 (Thermo Fisher Scientific) for 20 min at 4°C. The stained cells were washed, fixed with 4% paraformaldehyde, and analyzed on an Attune NxT flow cytometer (Thermo Fisher Scientific). To detect frequencies of IFN-γ^+^ cells, splenocytes were obtained from infected mice at day 10 postinfection. The cells were restimulated with inactivated IAV or SARS-CoV-2 (5,000 TCID_50_/mL) at 37°C for 5 h in the presence of monensin and brefeldin A (Thermo Fisher Scientific). The cells were washed, blocked with CD16/CD32 Fc Ab (BD Biosciences), and stained with APC anti-CD3 (clone 17A2), anti-CD4 (clone RM4-5), and PE-Cy7 anti-CD8 (clone 53-6.7) (Biolegend) for 30 min at 4°C followed by staining with fixable viability Dye eFluor 780 for 30 min at 4°C. The cells were fixed and permeabilized using the intracellular fixation and permeabilization buffer set (Thermo Fisher Scientific) and then stained with phycoerythrin (PE) anti-IFN-γ (clone XMG1.2, Invitrogen) for 1 h at room temperature (RT). The cells were washed and subjected to flow cytometric analysis. The data were then analyzed using FlowJo software (Tree Star, Ashland, USA).

### Viral titer determination.

Lung homogenates were prepared by homogenizing whole lung samples with beads in 0.7 mL virus culture media containing 1% PS using a homogenizer (Qiagen, Hilden, Germany). The lung homogenates were obtained by centrifuging the samples three times at 12,000 rpm for 10 min. MDCK cells or Vero cells were seeded in 96-well flat-bottom plates at 3 × 10^4^ cells per well. To determine influenza virus titers, confluent MDCK cells were washed twice with PBS and infected with lung homogenates or BALF, which was 10-fold serially diluted in EMEM containing 1% PS, at 37°C for 1 h. The cells were washed twice with PBS and incubated with EMEM containing 1 μg/mL l-(tosylamido-2-phenyl) ethyl chloromethyl ketone (TPCK)-trypsin (Worthington Biochemical, NJ, USA) and 1% PS at 37°C and 5% CO_2_ for 72 h. To determine SARS-CoV-2 titers, confluent Vero cells were washed twice with PBS and infected with lung homogenates or BALF, which was 10-fold serially diluted in DMEM containing 1% PS at 37°C for 1 h. The cells were washed twice with PBS and incubated with DMEM containing 0.4 μg/mL TPCK-trypsin and 1% PS at 37°C and 5% CO_2_ for 4 days. The appearance of the cytopathic effect of the virus was recorded daily, and the TCID_50_ was calculated according to Reed-Muench method and expressed as log_10_ TCID_50_/mL.

### Measurement of inflammatory cytokines and chemokines in BALF and serum.

A set of inflammatory cytokines and chemokines were measured in BALF and serum (for cytokines only) of each group of mice using the LEGENDplex mouse inflammation panel or LEGENDplex mouse proinflammatory chemokine panel kits (Biolegend, San Diego, USA) according to the manufacturer’s instructions. Briefly, BALF and serum were incubated with the assay buffer and beads in 96-well V-bottom plates for 2 h at room temperature (RT). After washing twice, the plates were incubated with detection Abs for 1 h at RT followed by incubation with streptavidin-PE (SA-PE) for 30 min at RT. After washing, the samples were acquired on an Attune NxT Flow cytometer (Thermo Fisher Scientific, Waltham, USA). The data were analyzed using the Biolegend LEGENDplex data analysis and Dongle software.

### Histopathological assay.

Whole-lung samples were harvested from mock and virus-infected mice at 7 dpi from the primary virus infection. The samples were fixed in 10% neutral-buffered formalin and embedded in paraﬃn. Deparaﬃnized sections were stained with hematoxylin and eosin (H&E). The stained sections were viewed and captured using an IX71 (Olympus, Tokyo, Japan) microscope with DP controller software.

### Enzyme-linked immunosorbent assay (ELISA).

Levels of IgG antibodies against IAV and SARS-CoV-2 were measured in sera of infected mice collected at day 10 postinfection. Briefly, 96-well Immuno plates (SPL Life Science, Gyeonggi-do, South Korea) were coated overnight at 4°C with 500 TCID_50_/well of inactivated IAV or SARS-CoV-2 in a carbonate solution. The plates were blocked with 5% skim milk in PBS for 2 h at 37°C and were washed with 0.05% Tween 20 in PBS. The plates were then incubated with the diluted sera (1:50 to 1:6,400) for 2 h at 37°C followed by incubation with horseradish peroxidase (HRP)-conjugated anti-mouse IgG (1:3000) (Jackson ImmunoResearch Laboratories, Inc., PA, USA) for 1 h at 37°C. The reactions were developed using *o*-phenylenediamine dihydrochloride for 30 min and stopped with 3 M HCl. The absorbance of the samples was measured at 450 nm using an ELISA plate reader (Molecular Devices, San Jose, USA).

### Serum neutralizing antibody (NAb) assay.

To evaluate serum NAb (SNAb) titers against IAV and SARS-CoV-2, sera were collected from the infected mice, heat inactivated at 56°C for 30 min, and 2-fold serially diluted (1:10 to 1:1280) for the SNAb assay. The diluted sera were incubated with an equal volume of either IAV or SARS-CoV-2 (100 TCID_50_) at 37°C for 1 h followed by incubation with confluent MDCK or Vero cells, respectively. After 1 h of incubation, the MDCK cells were washed and incubated with EMEM containing 1 μg/mL TPCK-trypsin and 1% PS at 37°C for 3 days, while the Vero cells were washed and incubated with DMEM containing 0.4 μg/mL TPCK-trypsin and 1% PS at 37°C for 4 days. The appearance of the cytopathic effect of the virus was recorded daily. The SNAb titers against IAV and SARS-CoV-2 were recorded at the indicated time points.

### Statistical analysis.

Statistical analysis was performed by two-way analysis of variance (ANOVA) with a Tukey test for multiple comparisons using GraphPad Prism 9.1.2 or an unpaired Student’s *t* test. A *P* value of ≤0.05 was considered significant.
