# Supplementary material for: Coinfection with SARS-CoV-2 and Influenza A Virus Increases Disease Severity and Impairs Neutralizing Antibody and CD4+ T Cell Responses
Source: J Virol. 2022 Mar 23;96(6):e01873-21. doi: 10.1128/jvi.01873-21 (PMC8941868; doi:10.1128/jvi.01873-21)
Supplement: Supplemental file 1 — Fig. S1 and S2. Download jvi.01873-21-s0001.pdf, PDF file, 0.5 MB [file jvi.01873-21-s0001.pdf]

Supplemental Material

Supplemental Figure 1. Coinfection of SARS-CoV-2 and IAV prolongs virus persistence in BALF.

Supplemental Figure 2. Coinfection of SARS-CoV-2 and IAV increases lung injury

Supplemental Figure 1

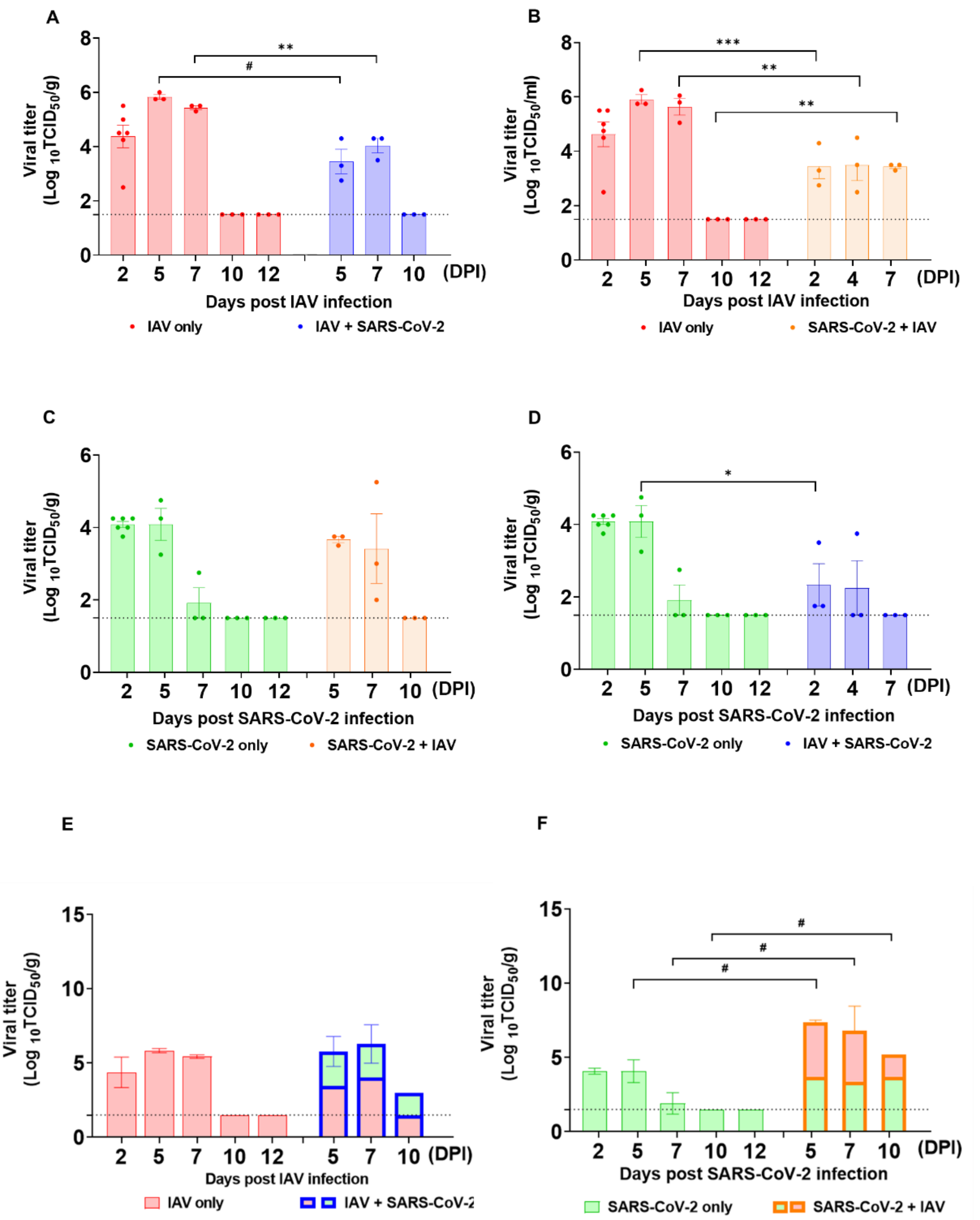

Supplemental Figure 1. Coinfection of SARS-CoV-2 and IAV prolongs virus persistence in BALF. K18-hACE2 mice were infected with either IAV or SARS-CoV-2 on day 0 and for coinfection groups, three days later the mice were infected with the other virus. At 2, 5, 7, 10, and 12 dpi in single infection groups and post-primary virus infection in coinfection groups (while 2, 4, 7 dpi post-secondary infection in coinfection groups would represent the 5, 7, 10 dpi), infectious IAV virus titers (A and B) and infectious SARS-CoV-2 titers (C and D) were measured in BALF of infected mice. Total viral load (sum of infectious IAV and SARS-CoV-2) (E and F) in BALF at the indicated time points. Data are shown as $log_{10}TCID_{50}$ and presented as mean ± SEM. Dashed lines indicate the limit of detection. Statistical significance was determined by two-way ANOVA/Tukey; *P < 0.05, **P < 0.01, ***P < 0.001, and #P < 0.0001.

Supplemental Figure 2

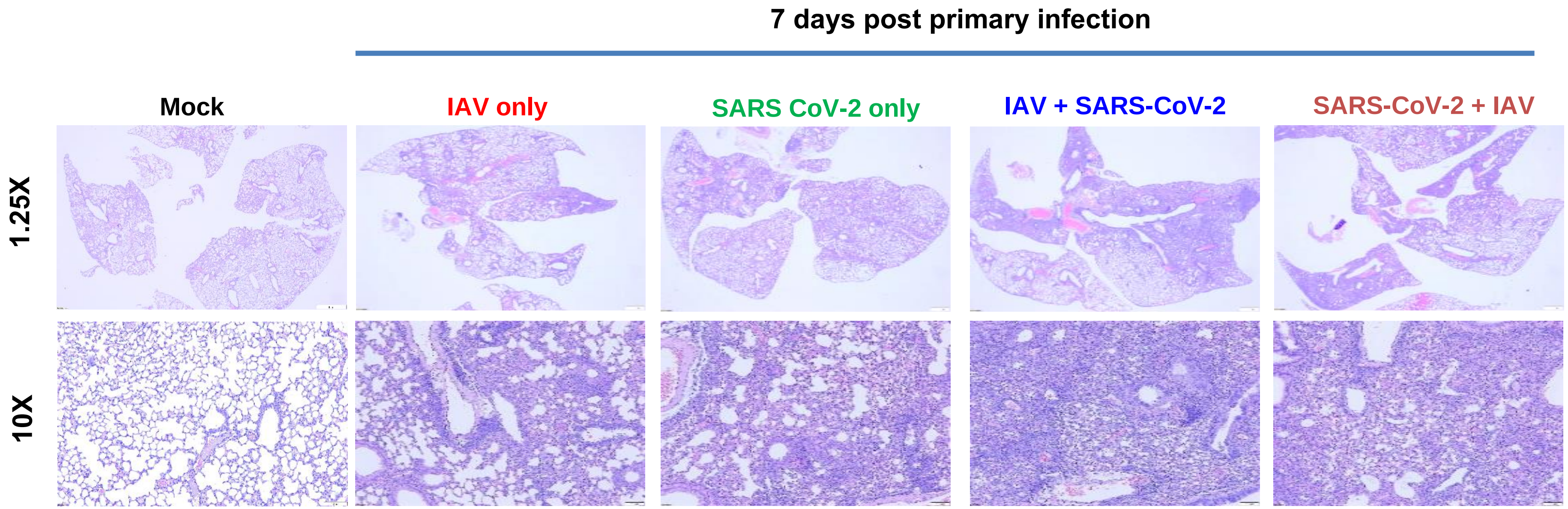

Supplemental Figure 2. Coinfection of SARS-CoV-2 and IAV increases lung injury. K18-hACE2 mice were infected with either IAV or SARS-CoV-2 on day 0. For coinfection groups, three days after primary infection mice were infected with the second virus. Hematoxylin and eosin staining of lung sections from infected mice at 7 days post primary infection. Images show 1.25X and 10X magnification, with scale bars, 1 mm and 100 µm, respectively.
